# Expression characterization of the herbicide tolerance gene Aryloxyalkanoate Dioxygenase (*aad-1*) controlled by seven combinations of regulatory elements

**DOI:** 10.1186/s12870-018-1227-3

**Published:** 2018-01-15

**Authors:** Delkin O. Gonzalez, Jeff B. Church, Andrew Robinson, James P. Connell, Megan Sopko, Boyd Rowland, Kristina Woodall, Cory M. Larsen, John P. Davies

**Affiliations:** 1 Dow AgroSciences, LLC, 9330 Zionsville Rd, Indianapolis, IN 46268 USA; 2 Current address: Purdue University College of Pharmacy, 575 Stadium Mall Drive, West Lafayette, IN 47907 USA

**Keywords:** Regulatory elements, Gene expression, Herbicide tolerance, Aryloxyalkanoate dioxygenase, Intron, Transcript, Protein

## Abstract

**Background:**

Availability of well characterized maize regulatory elements for gene expression in a variety of tissues and developmental stages provides effective alternatives for single and multigene transgenic concepts. We studied the expression of the herbicide tolerance gene aryloxyalkanoate dioxygenase (*aad-1*) driven by seven different regulatory element construct designs including the ubiquitin promoters of maize and rice, the actin promoters of melon and rice, three different versions of the Sugarcane Bacilliform Badnavirus promoters in association with other regulatory elements of gene expression.

**Results:**

Gene expression of *aad-1* was characterized at the transcript and protein levels in a collection of maize tissues and developmental stages. Protein activity against its target herbicide was characterized by herbicide dosage response. Although differences in transcript and protein accumulation were observed among the different constructs tested, all events were tolerant to commercially relevant rates of quizalafop-P-ethyl compared to non-traited maize under greenhouse conditions.

**Discussion:**

The data reported demonstrate how different regulatory elements affect transcript and protein accumulation and how these molecular characteristics translate into the level of herbicide tolerance. The level of transcript detected did not reflect the amount of protein quantified in a particular tissue since protein accumulation may be influenced not only by levels of transcript produced but also by translation rate, post-translational regulation mechanisms and protein stability. The amount of AAD-1 enzyme produced with all constructs tested showed sufficient enzymatic activity to detoxify the herbicide and prevent most herbicidal damage at field-relevant levels without having a negative effect on plant health.

**Conclusions:**

Distinctive profiles of *aad-1* transcript and protein accumulation were observed when different regulatory elements were utilized in the constructs under study. The ZmUbi and the SCBV constructs showed the most consistent robust tolerance, while the melon actin construct provided the lowest level of tolerance compared to the other regulatory elements used in this study. These data provide insights into the effects of differing levels of gene expression and how these molecular characteristics translate into the level of herbicide tolerance. Furthermore, these data provide valuable information to optimize future designs of single and multiple gene constructs for maize research and crop improvement.

**Electronic supplementary material:**

The online version of this article (doi: 10.1186/s12870-018-1227-3) contains supplementary material, which is available to authorized users.

## Background

The use of herbicide tolerance genes in genetically modified crops is a powerful practice for implementation of effective weed control programs. Several different herbicide tolerance genes and modes of action have been used in crops such as soybean, cotton, maize and canola [1]. The aryloxyalkanoate dioxygenase (*aad-1*) gene is a herbicide tolerance gene that encodes an enzyme which detoxifies aryloxyphenoxypropionate herbicides via an α-ketoglutarate dependent dioxygenase reaction [2]. Plants transformed with the *aad-1* gene are able to detoxify the herbicide quizalofop-P-ethyl and 2,4-dichlorophenoxyacetic acid at commercial rates of application showing no herbicidal effects.

Herbicide tolerance genes are often expressed under the control of constitutively expressed regulatory elements so that the herbicides can be applied at all growth stages without damage to the plant. These constitutive plant regulatory elements are characterized by continuous and consistent activity during development in most or all plant tissues [3] and are found in numerous transgenic products [4]. However, repeated use of the same promoter within a construct can lead to gene silencing [5–7], which can pose a problem in constructs requiring multiple genes. Evaluation and characterization of multiple constitutive regulatory elements controling the expression of herbicide tolerance genes will therefore provide additional alternatives for transgenic product development and for the optimized design of multigene constructs.

Here, we report the characterization of the aryloxyalkanoate dioxygenase gene expression (*aad-1*) under the control of seven different regulatory elements, including the ubiquitin promoters from maize [8] and rice [9], Actin promoters from melon [10] and rice [11] and three different versions of the Sugarcane Bacilliform Badnavirus (SCBV) promoters: SCBV-from the Ireng Maleng (IM) genome [12], SCBV from the IM genome plus the maize ubiquitin 1 intron (I) and the SCBV from the Mor (UM) genome [13]. Transcript and protein abundance were examined in trangenic events generated with each construct in several tissues at different stages of development. Events containing the ZmUbi promoter driving expression of the *aad-1* gene served as a benchmark due to its extensive use in characterization studies in maize [8, 14] and other plant species such as rice, sugarcane and palm oil [15–17].

## Results

### Transcript abundance analysis

**Fig. 1:**
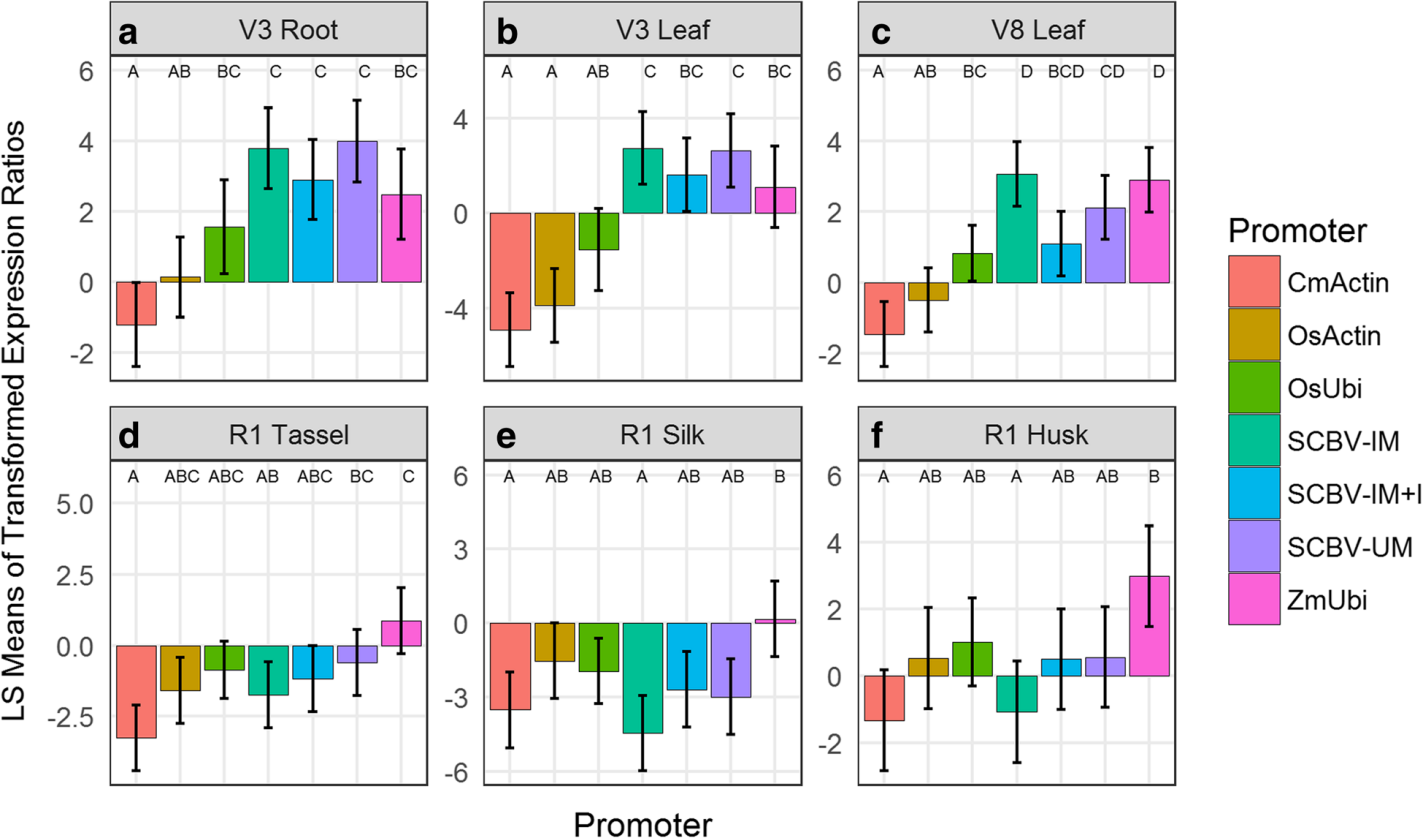
Effect of promoter on relative AAD-1 transcript abundance by tissue and developmental stage. Least square mean estimates of the log2 transcript-to-reference ratios are shown, as well as the 95% confidence intervals and the groupings (e.g. AB) from a post-hoc Tukeys HSD (α = 0.05). Panel **a**: Root tissues collected at vegetative Stage 3. Panel **b** and **c**: Leaf tissues collected at vegetative stages 3 and 8 respectively. Panels **d**, **e** and **f**: Tissues collected at the reproductive stage 1 for Tassel, Silk and Husk respectively

All events containing the melon actin (CmActin), rice actin (OsActin), rice ubiquitin (OsUbi), Sugarcane Bacilliform Badnavirus Irene Maleng genome (SCBV-IM), Sugarcane Bacilliform Badnavirus Irene Maleng genome plus intron (SCBV-IM + I) and Sugarcane Bacilliform Badnavirus Mor genome from University of Minessota (SCBV-UM) promoters and regulatory elements driving the *aad-1* herbicide resistance gene accumulated *aad-1* transcripts (Fig. 1, Additional file 1). However, differences in *aad-1* transcript accumulation between events having these transgenes and events generated with ZmUbi::*aad-1* were observed. Figure 1a-e) shows that events carrying the SCBV-IM, SCBV-IM + I and SCBV-UM viral derived promoters driving *aad-1* accumulated levels of the *aad-1* transcript comparable to events containing the ZmUbi promoter driving *aad-1* in most tissues examined; an exception is that events containing SCBV-IM::*aad-1* had significantly less *aad-1* transcript in tassel, silk and husk tissues at the 1st reproductive (R1) growth stage [18] than events containing ZmUbi::*aad-1.* Events containing CmActin::*aad-1* accumulated significantly lower levels of the *aad-1* transcript than events containing ZmUbi::*aad-1* in all of the tissues examined. Also, relative to ZmUbi::*aad1* events, OsActin::*aad-1* and OsUbi::*aad-1* containing events showed no difference in the level of *aad-1* transcript accumulation in roots of plants at the 3rd vegetative growth (V3) stage [18] and the tassel silk and husk at R1. However, in leaf tissues, the OsActin::*aad-1* events accumulated significantly lower levels of *aad-1* transcript than ZmUbi events at V3 and the eighth vegetative (V8) stage [18] while OsUbi::*aad-1* events showed significantly lower levels in leaves at V8.

### Protein abundance analysis

**Fig. 2:**
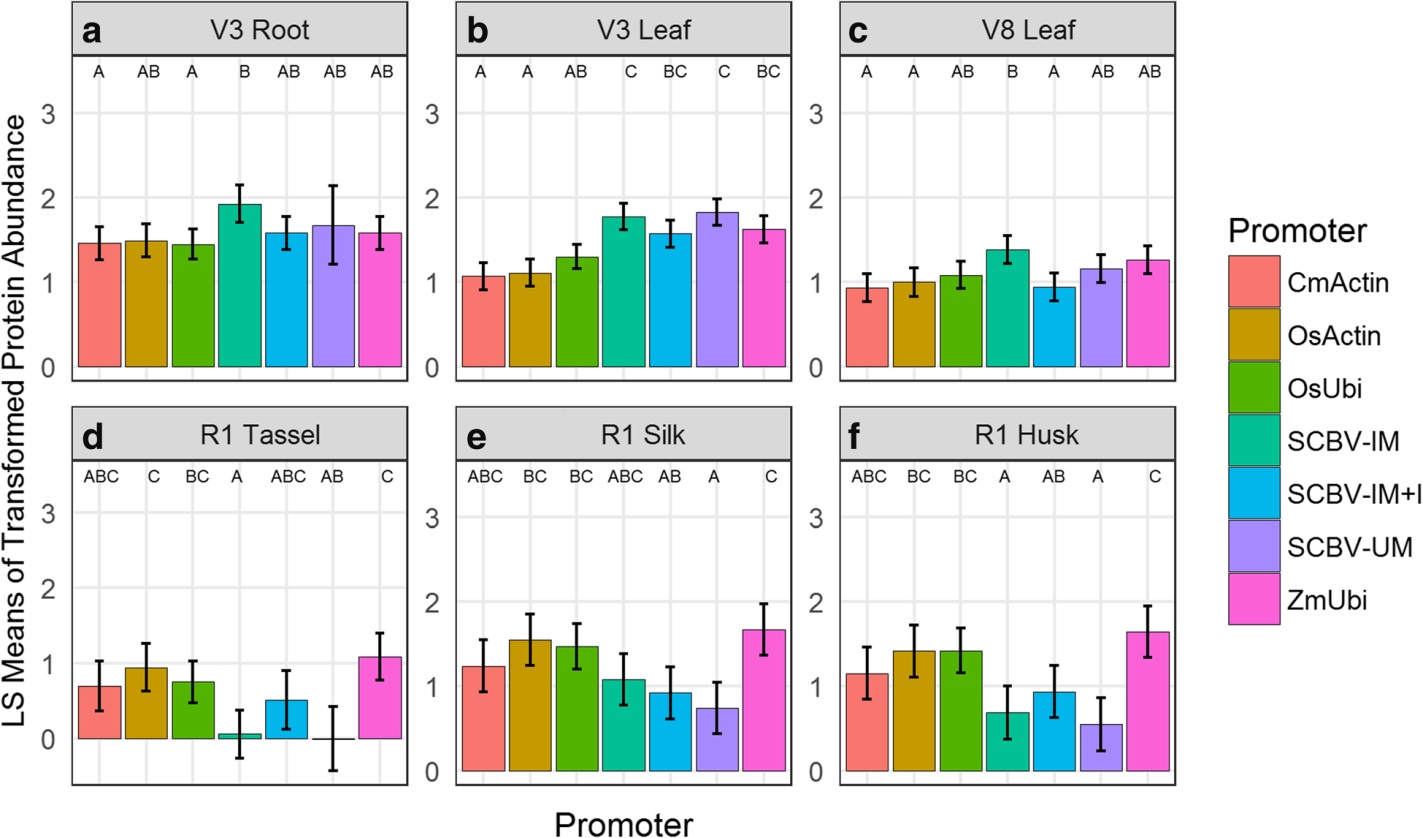
Effect of promoter on AAD-1 protein abundance values by tissue and developmental stage. Least square mean estimates of the log2 accumulation values are shown, as well as the 95% confidence intervals and the groupings from a post-hoc Tukeys HSD (α = 0.05). Panel **a**: Root tissues collected at vegetative Stage 3. Panel **b** and **c**: Leaf tissues collected at vegetative stages 3 and 8 respectively. Panels **d**, **e** and **f**: Tissues collected at the reproductive stage 1 for Tassel, Silk and Husk respectively

The AAD-1 protein accumulation data (Additional file 2) show some differences in protein accumulation in events containing the tested regulatory elements and those containing ZmUbi. Figure 2 shows a summary of the levels of AAD-1 protein accumulated in root (V3), leaf (V3 and V8), husk (R2), silk (R1) and tassel (R1) tissues in events generated from the different constructs. Events containing the SCBV-derived promoters (SCBV-IM, SCBV-IM + I and SCBV-UM) accumulated levels of AAD-1 protein that were not significantly different that those accumulated in ZmUbi containing events in most tissues (Fig. 2). Exceptions are that relative to ZmUbi, events containing SCBV-IM and SCBV-UM driving the *aad-1* gene accumulated significantly less AAD-1 protein in tassel and husk tissues (Fig. 2d, f) while events containing SCBV-IM + I and SCBV-UM promoters showed less AAD-1 protein accumulation. Events containing CmActin::*aad-1* and OsActin::*aad-1* accumulated levels of AAD-1 protein comparable to events containing ZmUbi::*aad-1* in all tissues except V3 leaves (Fig. 2b). Events carrying the OsUbi::*aad-1* showed no difference in levels of AAD-1 protein accumulation compared with ZmUbi::*aad-1* events in all tissues tested. The SCBV-derived regulatory elements without the maize Ubiquitin 1 intron (SCBV-IM and SCBV-UM) accumulated significantly lower levels of AAD-1 protein in tassel tissues relative to ZmUbi events (see Fig. 2d).

### Herbicide tolerance analysis

**Fig. 3:**
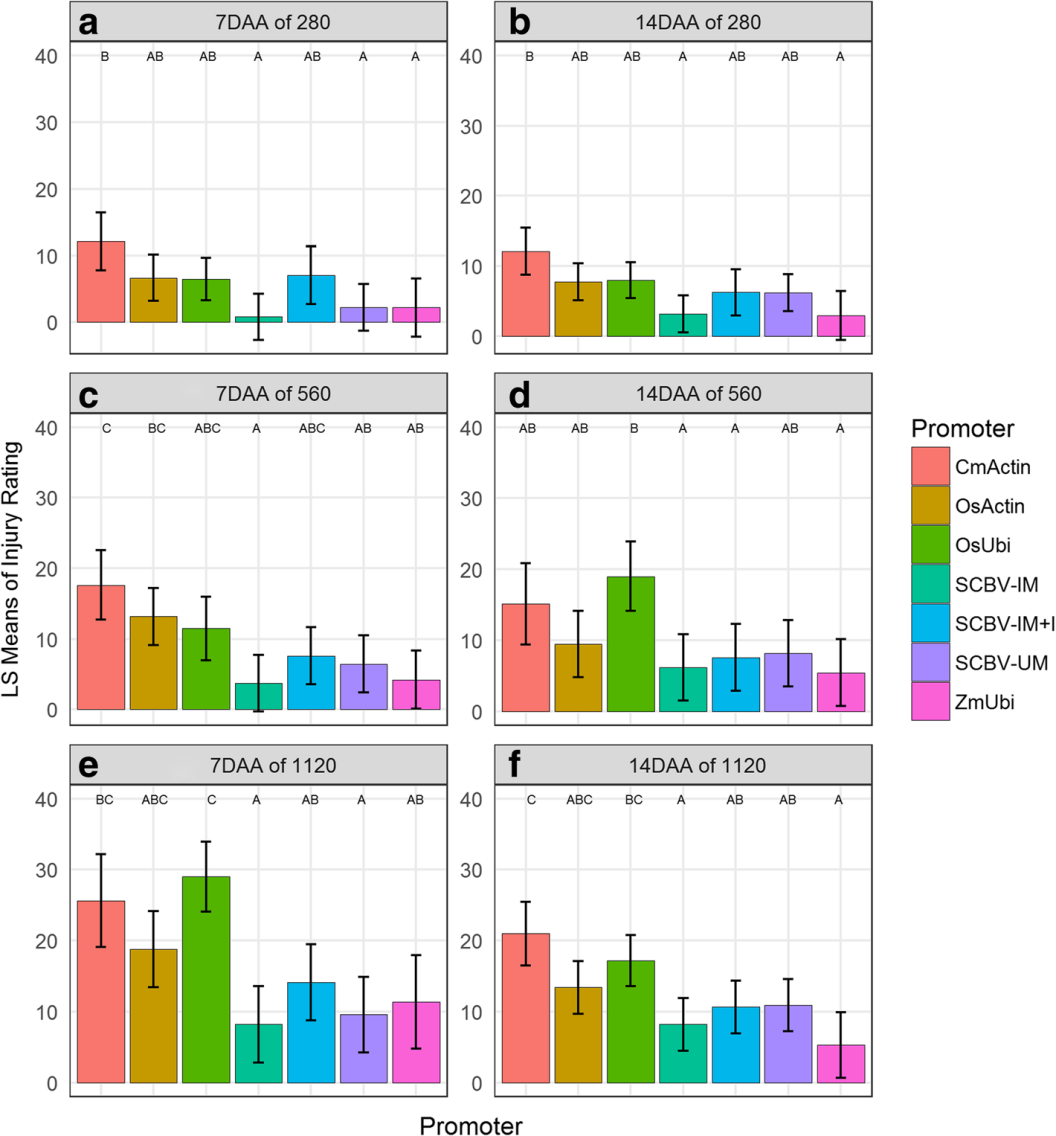
Effect of promoter on vegetative injury ratings by application rate. Three rates were used: 280, 560 and 1120 g ae ha^−1^. Plant injury was visually scored at 7 or 14 days after application (DAA). Least square mean estimates of the injury percentages are shown, as well as the 95% confidence intervals and the groupings from a post-hoc Tukeys HSD (α = 0.05). Panel **a**,**b**: Applied rate of 280 g ae ha^-1^ at 7 and 14 DAA respectively. Panel **c**,**d**: Applied rate of 560 g ae ha^-1^ at 7 and 14 DAA respectively. Panel **e**,**f**: Applied rate of 1120 g ae ha^-1^ at 7 and 14 DAA respectively

To test the effectiveness of these constructs to confer tolerance to quizalafop-P-ethyl, hemizygous transgenic plants were grown to the V3 stage, sprayed with the herbicide at a rate from 280 to 1120 g equivalent per hectare (g ae ha^−1^) and evaluated for damage at 7 and 14 days after application (DAA). The lowest rate tested of 280 g ae ha^−1^ is approximately four times (4×) the use rate to control non-traited corn. The results obtained (Fig. 3 and Additional file 3) at the 280 g ae ha^−1^ rate at both 7 and 14 DAA show that events containing six of the seven constructs showed less than 10% visual injury while the seventh (CmActin::*aad-1* containing events) showed less than 15% visual injury (Fig. 3a, b). Also at this rate, events containing SCBV-IM::*aad-1*, SCBV-IM + I::*aad-1*, SCBV-UM::*aad-1*, OsUbi::*aad-1* and OsActin::*aad-1* showed no difference in herbicide damage compared with events containing ZmUbi::*aad-1* both at 7 and 14 DAA while events containing the CmActin::*aad-1* consistently showed significantly greater herbicide damage than events containing ZmUbi::*aad1*. These results are consistent with V3 leaf protein accumulation data; application of quizalafop-P-ethyl on leaves was performed at the V3 stage and events containing the CmActin::*aad-*1 construct accumulated significantly lower levels of AAD-1 protein than events containing the ZmUbi promoter.

To better differentiate the effect conferred by the expression of the *aad-1* gene under control of different promoters, the transgenic plants were treated with quizalafop-P-ethyl at 560 and 1120 g ae ha^−1^, these rates are approximately eight (8×) and sixteen (16×) times the rate to control non-traited corn, respectively. In the 560 g ae ha^−1^ treatment, events containing SCBV-IM::*aad-1*, SCBV-IM + I::*aad-1*, SCBV-UM::*aad-1*, and OsActin::*aad-1* showed tolerance to the herbicide and no significant difference was observed relative to events containing the ZmUbi::*aad1* constructs (Fig. 3c, d). However, events containing CmActin::*aad-1* (at 7 DAA) and OsUbi::*aad-1* (at 14 DAA) showed significantly more damage than events containing ZmUbi::*aad-1* construct. In the 1120 g ae ha^−1^ treatment, overal herbicide damage observed was greater, however events containing the SCBV-derived promoters performed as well as the ZmUbi::*aad-1* events at both 7 and 14 DAA (Fig. 3e, f). The OsUbi::*aad-1* (at 7 DAA) and the CmActin::*aad-1* (at 14 DAA) events showed significantly more damage than the ZmUbi::*aad-1* events.

## Discussion

Agricultural biotechnology has produced engineered crops that tolerate a number of broad spectrum herbicides, enabling the implementation of efficient weed control programs [1, 19]. Furthermore, the incorporation of herbicide tolerant crops into farming systems has enabled the optimization of tillage practices and associated improvements in soil quality [20]. In order to delay the development of herbicide resistance in weeds, novel herbicide resistance genes and modes of action need to be integrated into weed management programs. Use of products containing multiple herbicide tolerance traits is currently a common practice to manage herbicide tolerant weeds in transgenic crops [21]. At the molecular level, understanding the expression of herbicide tolerance genes is necessary for the development of single and stacked traits that will fulfill the needs of farmers. The results obtained in this study provide insights into the effects of differing levels of expression on herbicide tolerance and product performance. The data presented here demonstrate how different regulatory elements affect transcript and protein accumulation and how these molecular characteristics translate into the level of herbicide tolerance.

Transgenic events containing the SCBV- viral derived promoters showed levels of *aad-1* transcript and AAD-1 protein accumulation that were not statistically different from events containing the ZmUbi promoter in leaf and root tissues. Events containing SCBV-IM + I and SCBV-UM showed levels of *aad-1* transcript not statistically different than the ZmUbi::*aad-1*-containing events in silk and tassel tissues while events containing the SCBV-IM promoter without the ZmUbi intron showed lower *aad-1* transcript accumulation in tassel tissues.

Events generated with the SCBV-IM promoter construct produced statistically comparable *aad-1* transcript abundance levels in all three vegetative tissues relative to the levels observed with the ZmUbi promoter while in all three reproductive tissues (tassel, silk and husk) showed significantly lower *aad-1* transcript accumulation relative to ZmUbi. The data suggests that the molecular mechanisms required to activate the SCBV-IM promoter and produce a transcript is highly efficient in the vegetative tissues relative to the reproductive tissues at the developmental stages tested. A very similar pattern was observed at the protein accumulation level in all tissues tested except silk where the SCBV-IM promoter delivered comparable levels of AAD-1 protein relative to the ZmUbi promoter. This pattern of expression could be leveraged to generate transgenic maize expressing genes of interest at higher levels in vegetative tissues and lower levels in some reproductive tissues.

Many introns have been reported to cause an increase in transcript accumulation when they are included in transgenic designs [22, 23]. The first intron from the maize Ubiquitin 1 gene has been specifically cited as an element that can cause increased expression of transgenes [24–26]. In this work, the SCBV IM promoter was tested with and without the maize Ubiquitin 1 intron to drive expression of the *aad-1* gene (the SCBV-IM::*aad-1* and SCBV-IM + I constructs). The data presented here do not show statistically significant differences in transcript accumulation associated with the presence of the ubiquitin 1 intron.

Several differences between AAD-1 protein and *aad-1* transcript accumulation data relative to ZmUbi were observed. For example, in V3 roots as well as in V3 and V8 leaves, there was a significant difference in *aad-1* transcript levels between events utilizing CmActin and those using ZmUbi, SCBV-IM, SCBV-IM + I and SCBV-UM promoters (Fig. 1a-c). However, AAD-1 protein accumulation in the same tissues showed a more variable profile between events containing the same constructs. In root tissues at V3 and leaves at V8 no significant differences were detected between events carrying the CmActin promoter and those using ZmUbi, SCBV-IM + I and SCBV-UM promoters while the SCBV-IM showed significantly different AAD-1 protein accumulation. Also in leaf tissues at V3 AAD-1 protein accumulation was significantly different between events utilizing CmActin and those using ZmUbi, SCBV-IM, SCBV-IM + I and SCBV-UM promoters (Fig. 2a-c). These observations suggest that, at least in this study, the level of transcript accumulated in most tissues does not necessarily reflect the amount of protein accumulated in a particular tissue. Protein accumulation levels may be influenced by the level of transcript present but also by the *aad-1* rate of translation, post-translational regulation mechanisms and protein stability.

The herbicide tolerance data provides additional information about protein function and efficiency related to specific biochemical mechanisms of herbicide detoxification. In this particular case, we determined that the amount of aryloxyalkanoate dioxygenase enzyme produced had sufficient enzymatic activity to detoxify the herbicide and prevent most herbicidal damage at field-relevant levels. Our results demonstrated that when quizalafop-P-ethyl was applied at four times (4×) the use rate to control non-traited corn, all events containing these constructs showed robust protection against herbicide damage (expressed as ˂ 15% average visual injury) at 7 or 14 DAA. These observations suggest that even the lowest amount of AAD-1 protein accumulation detected in the CmActin::*aad-1* containing events is enough to confer robust tolerance to herbicide applications at commercially relevant rates. In addition, transgenic plants with high levels of accumulated aryloxyalkanoate dioxygenase enzyme, appeared healthy and no major differences were observed relative to the negative control.

All the promoters evaluated in this study effectively drove expression of the *aad-1* gene and can be used for transgenic plant production in maize. These elements can be used to modulate the specific production of proteins in maize associated not only with herbicide tolerance functions but also a broad selection of relevant plant related applications such as pathogen resistance and nutrient absorption among others.

Constructs built with the regulatory elements included in this study can effectively express proteins with variable and specific efficiencies as well as tissue and developmental requirements in maize. For example, events containing the SCBV-IM and the ZmUbi promoters produced comparable levels of transcript and protein in leaf and root samples while in tassel and husk tissue transcripts and protein were lower in the SCBV-IM events. Therefore, if high level expression for a particular gene with a different metabolic function and activity is desired in leaves and roots, but not in male reproductive tissues, the SCBV IM may be a good choice. On the other hand, events containing the CmActin promoter had low *aad-1* transcript and protein accumulation in most tissues and developmental stages tested. This promoter may be suitable for expressing specific genes where high levels of protein accumulation may be detrimental. Having a diverse resource of well-characterized regulatory elements provides opportunities to optimize the design of multigenic constructs that require regulated expression of multiple genes in different tissues and developmental stages.

## Conclusions

Analysis of transcript abundance of *aad-1* by real time quantitative polymerase chain reaction (RT-qPCR) in transgenic events demonstrated that different version of the SCBV regulatory element combinations tested produced dissimilar profiles of *aad-1* expression. Events containing the SCBV IM + intron and SCBV UM regulatory elements produced comparable levels of *aad-1* transcript accumulation than the ZmUbi promoter in all tissues tested. The SCBV-IM::*aad-1* regulatory elements produced significantly less *aad-1* transcript in tassel, silk and husk tissues than events containing ZmUbi::*aad-1*. Additional differences in *aad-1* transcript accumulation were observed when the CmActin, OsActin and OsUbi regulatory elements were compared with ZmUbi events; events containing the CmActin accumulated significantly lower levels of the *aad-1* transcript in all tissues examined, and events containing the OsActin regulatory elements showed significantly lower levels of transcript accumulation in one or more of the leaf samples.

Despite observing significant differences in *aad-1* transcript accumulation, few differences in AAD-1 protein accumulation were observed between events with the different constructs. Events containing the SCBV-derived promoters showed levels of AAD-1 protein in root and leaf tissues that were not statistally different than those of events containing the ZmUbi promoter. However, some events containing the SCBV-derived promoters showed statistically lower levels in tassel, silk and husk tissues during the maize reproductive phase. The events containing the CmActin, OsActin and OsUbi showed few differences in AAD-1 protein accumulation when compared with the ZmUbi::*aad-1* containing events despite showing significant differences in *aad-*1 transcript accumulation. The results presented here demonstrate that with the technologies used, transcript accumulation data does not necessarily reflect the amount of protein accumulated in a particular tissue.

The herbicide tolerance study showed that transgenic events tested for each construct at the different rates conferred tolerance to elevated levels of quizalafop-P-ethyl. Although significant differences in transcript and protein abundance in V3 leaf tissues were observed, all constructs provided robust tolerance to the 4X, 8X and 16X field rates tested in V3 stage plants. Transgenic plants with high levels of accumulated aryloxyalkanoate dioxygenase enzyme, appeared healthy and no major differences were observed relative to the negative control. The CmActin promoter provided the lowest level of tolerance compared to the other promoters used in this study, while the ZmUbi and the SCBV promoters showed the most consistent robust tolerance.

All the regulatory element combinations assessed in this study effectively drove expression of the *aad-1* gene and can be used for transgenic plant production in maize. This diverse resource of well-characterized regulatory elements in maize provides a useful molecular tool to optimize multigenic construct design and the modulation of expression of multiple genes in different tissues and developmental stages.

## Methods

Transgenic maize events were generated via *Agrobacterium* transformation with vectors containing the herbicide tolerance gene aryloxyalkanoate dioxygenase (*aad-1*) under the control of different genetic regulatory elements. The effects of the regulatory elements were assayed by examining *aad-1* transcript and protein accumulation by RT-qPCR and ELISA respetively, in several tissues during the maize vegetative and reproductive growth phases. To test the effectiveness of these constructs to confer tolerance to quizalafop-P-ethyl, hemizygous transgenic plants were grown to the V3 stage, sprayed with the herbicide and visually examine plant injury at two different time points.

### Regulatory elements studied

#### Sugarcane bacilliform Badnavirus (SCBV)

The first report for the Sugarcane Bacilliform Badnavirus (SCBV) promoter came from the University of Minnesota in 1998 [13]. They describe a region of 1421 nucleotides of the SCBV genome (Sugarcane bacilliform Mor virus GenBank accession number M89923.1) spanning nucleotides 5999–7420 with promoter activity tested in *Avena sativa* and *Arabidopsis thaliana*. Another SCBV promoter was identified from the genome of Sugarcane Bacilliform IM virus (Isolate Ireng Maleng) [12] GenBank Accession AJ277091.1. This genome is 7687 bp in size and has 76% sequence identity to the Sugarcane Mor genome. A promoter region of 839 bp has been defined between positions 6758–7596 and has been previously characterized in maize [27]. There is a 260 bp region of 77% sequence identity between the 5′ end of the IM promoter (position 6758–7018) and positions 6706–6966 of the Mor genome.

**Fig. 4:**
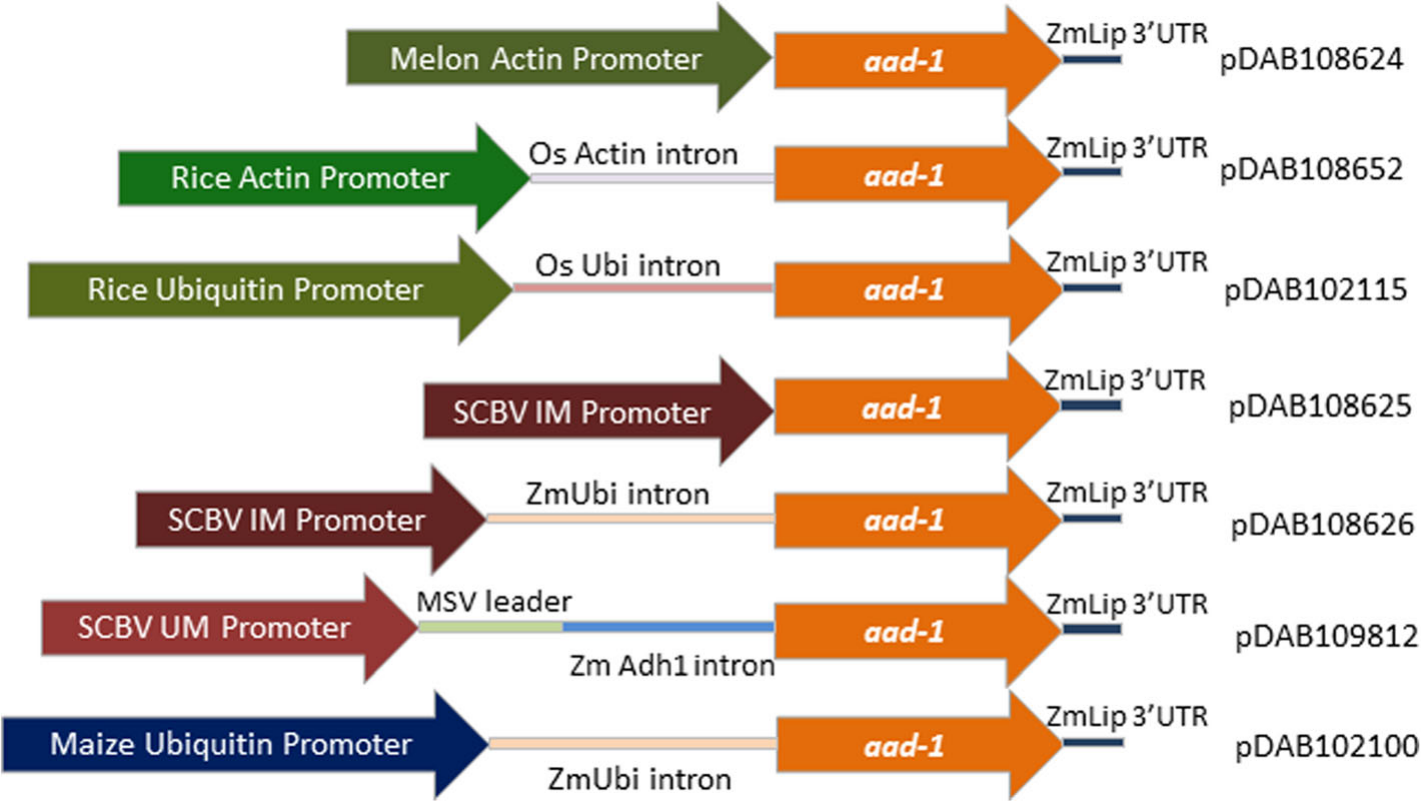
Schematic representation of the regulatory elements controlling the expression of the *aad-1* gene

Three constructs (Fig. 4), using the two available versions of the SCBV promoter driving the expression of the *aad-1 gene*, were available for this study. First, pDAB108625 contains the 839 bp SCBV IM promoter sequence (from 6758 to 7496 bp from the IM genome), driving the *aad-1* gene and using the maize lipase (ZmLip) 3′ untranslated region (ZmLip 3’ UTR). Another construct, pDAB108626, contains the same SCBV IM promoter as pDAB108625 with the addition of the ZmUbi exon and intron added to the 5’ UTR. The third construct, pDAB108912, contains the SCBV UM (from 5999 to 7420 bp of the Mor genome) the Maize Streak Virus (MSV) leader sequence and the alcohol dehydrogenase (Adh1) intron, driving the expression of the *aad-1* gene and using the ZmLip 3’UTR. All of these constructs contain a second gene expression cassette that includes the ZmUbi1 promoter and intron driving the expression of the *phialidium* yellow fluorescent protein (PhiYFP) reporter gene.

#### Melon Actin

The Melon Actin (CmActin) promoter data [10] showed higher transient levels of expression in onion cells relative to the CsVMV and the CaMV35S promoters. The construct used in this study (pDAB108624) was developed for testing in stable transgenic maize where the CmActin promoter (without intron,) is driving the expression of the *aad-1 gene* (Fig. 4). pDAB108624 also contains a second gene expression cassette that includes the ZmUbi1 promoter and intron driving the expression of the PhiYFP reporter gene.

#### Rice Actin

The construct used for this study (pDAB108652) contains a cassette with the Rice Actin 1 (OsActin) promoter [11] including the rice Actin 1 intron driving the expression of the *aad-1 gene* and the ZmLip 3’UTR (Fig. 4). This construct also contains a second gene expression cassette that includes the ZmUbi1 promoter and intron driving the expression of the PhiYFP reporter gene.

#### Rice Ubiquitin

The rice ubiquitin 3 (OsUbi) promoter [9] has been characterized as a high expression monocot promoter. For this study we used the construct pDAB102115 (Fig. 4) containing a cassette with the OsUbi promoter and intron driving the expression of the *aad-1 gene* and the ZmLip 3’ UTR. This construct also contains a second gene expression cassette that includes the ZmUbi1 promoter and intron driving the expression of the PhiYFP reporter gene.

#### Maize Ubiquitin

The maize Ubiquitin 1 (ZmUbi) promoter [8] was first described in 1992 and since then it has been one of the most frequently utilized constitutive promoters in maize. Construct pDAB102100 (Fig. 4) contains the ZmUbi1 promoter including the ZmUbi intron driving the *aad-1* gene using the ZmLip 3’UTR. Upstream of this cassette there is a second gene expression cassette that includes the ZmUbi1 promoter and intron driving the expression of the PhiYFP reporter gene.

#### Maize transformation

Experimental constructs were transformed into *Zea mays* via *Agrobacterium*-mediated transformation of immature embryos isolated from the inbred line, *Zea mays* c.v. B104. The method used is a modified version of previously reported methods [28, 29]. In summary, immature embryos of approximately 1.8 to 2.4 mm in length were isolated from *Zea mays* c.v. B104. Isolated embryos were then incubated with an *Agrobacterium* suspension media containing acetosyringone and the surfactant Break Thru S 233® at an Optical Density of 1.0 at 600 nm for 20–30 min and placed on co-cultivation medium, oriented scutellum-up for 3–4 days. Embryos were then transferred onto a selection-free medium containing antibiotics (10 mg/L rifampicin, 50 mg/L spectinomycin and 50 mg/L streptomycin) for 7 days to suppress *Agrobacterium* growth and begin callus formation. The calli were then placed on medium containing 100 nM Haloxyfop for 1 week and 500 nM Haloxyfop for 2 weeks. Following the selection step, calli were placed on selection medium containing plant growth hormones and 500 nM Haloxyfop for 7 days to begin somatic embryo germination. After 1 week of exposure to the plant growth hormone medium, calli were placed on a plant regeneration medium containing 500 nM Haloxyfop for selection. Plants typically form within 1–2 weeks after being transferred to the plant regeneration medium. Developed plantlets were isolated to plant growth medium and about 10–15 mg of leaf tissue was sampled for TaqMan genotyping analysis of selection marker *aad-1* gene. Between 20 to 30 simple events per construct were selected and transferred to the greenhouse facilities for transplantation to soil. All T0 events were de-tasseled and pollinated with *Zea mays* c.v. B104 pollen for T1 seed production.

### Maize transgenic T1 event selection, seed germination, transplantation

T1 seed from specific events was selected to be used in this study, based on transcript abundance data available from the corresponding T0 event. A total of six events per construct and 25 seeds per event (150 seeds per construct) were planted to ensure that 5 healthy hemizygote events were available for characterization. Glasshouse environmental conditions were set for a day temperature of 29 °C and a night temperature of 26 °C. Supplemental lighting was set for a 14:10 h day/night cycle. Seeds were germinated in a peat pot (Jiffy Poly-Pak™ 440) filled with commercial grade soil-less mix (Sunshine® Mix #2 / LB2). Seeds were sown 3.5 cm deep, one kernel per pot into pre-moistened media at field capacity. Plants were sampled for genotyping 7 DAP (days after planting).

A total of 98 plants at 22 DAP were selected for transplantation a traditional 5 gal plastic pot with a 50/50 mixture of Metro Mix® 360 and Profile Greens Grade™. A total amount of 1.78 kg of each Osmocote™ 19–6-12 and Ironite® 1–0-1 were added to each cubic meter of soil during the mixing stage prior to filling each pot. The 5 gal pots were saturated before transplanting. Each plant was transplanted at a depth that corresponded with the soil surface in the peat pot. The plants were transferred to the greenhouse and arranged in a 4 row randomized block.

#### Tissue sample collection

Samples were collected from root (V3), leaf (V3 & V8), tassel (R1), silk (R1) and husk (R2), tissues for transcript and protein abundance analysis. All samples for transcript abundance were collected in 96-well collection tube plates (Qiagen). Each sample tube was capped with a Micronic pierceable TPE cap, and then frozen for 3–5 s in liquid nitrogen. Sample tubes were transferred to a 96-well rack on dry ice, and then transferred to storage at −80 °C. Samples for protein analysis were collected in a similar manner, with the exception of silk, tassel and husk tissues, which were collected in 50 mL tubes (Fisher Scientific), then frozen on dry ice and transferred to storage at −80 °C. These samples were lyophilized and normalized based on dry weight for ELISA analysis.

#### Sample collection at V3

Leaf and root samples were collected at the V3 growth stage, 14 to 15 DAP. For the leaf tissue, a standard office hole puncher modified to hold a tube underneath for sample collection was used to punch one disc of leaf tissue into each tube of a 96-well collection tube plate. One leaf disc each for transcript and protein analysis was taken near the leaf tip.

Four root samples were collected from each plant. Two average sized white roots protruding from the bottom or sides of the pot, at least 2.5 cm in length, were sampled. Dissecting scissors were used to cut a sample of approximately 1 cm long from each root tip. Large clumps of dirt were removed from the samples, and each of the four root samples were placed in separate sample collection tubes.

#### Sample collection at V8

A second leaf sampling was performed when plants had reached the V8 growth stage, 41 DAP. Three samples were collected from each plant. For transcript abundance analysis, one leaf disc was sampled from near the middle of the uppermost collared leaf (approximately equidistant from the tip and collar) on one side of the midrib, and a duplicate backup sample was taken from the opposite side of the midrib. Protein results from the V3 leaf samples indicated that four leaf discs would be a more optimal sample size than the single punch used for V3. Four discs were collected adjacent to the punches for transcript analysis, two on each side of the midrib, and placed into a single collection tube for each plant.

#### Sample collection at R1 and R2

As individual plants reached the R1 silking stage, samples were collected from silk and tassel tissues. The tassels were bagged using a Midco Enterprises medium tassel bag the afternoon prior to the day of sample collection. The morning of sample collection the bags were removed from the plants individually as the sample was to be collected. Silk sample collection was begun with plants that had reached the R1 growth stage at 62 DAP. The shoot bag (Midco Enterprises) was removed from the developing ear and scissors were used to cut off the husk approximately 2.5 cm from the top of the developing ear. Silks were collected from the portion that was cut from the ear by removing the husk to reveal the silks. For transcript analysis, a target amount of five 1-cm pieces of silk, approximately 10–20 mg, was cut and placed into a collection tube. The remainder silk tissue was lyophilized and samples were normalized to 10 mg of dry tissue for ELISA analysis.

Tassel samples from the first side stalk were collected from plants once they had reached the R1 growth stage, beginning at 65 DAP. Anthers were removed, and then a 2 cm section, approximately 10–30 mg, was cut from the tip of the stalk using scissors. This section was cut into small pieces, approximately 2–4 mm in length, and the pieces were placed into a collection tube for transcript analysis. For protein analysis, the remainder of the cut stalk of tassel was lyophilized in a 50 ml tube. Approximately 1 in. of lyophilized tassel tissue was used for protein quantification by ELISA.

Samples were collected from husk tissue as plants reached the R2 blister stage, beginning at 71 DAP. The husk was split using a spatula and pulled back from the ear, and a section from the innermost husk was removed. Using the same method as for leaf tissue, a hole puncher was used to punch one disc of husk tissue into each collection tube for transcript analysis. For protein analysis, the remainder of the piece of husk was lyophilized and samples were normalized to 30 mg of dry tissue for ELISA analysis.

#### Zygosity of aad-1 transgene determined by qPCR

Hemizygous plants were identified for transplanting and further analysis. Hemizygous plants containing more than 2 copies of *aad-1* were excluded from the study. Plants were tested for the presence of the *aad-1* gene by qPCR analysis. Copy number of the *aad-1* gene was determined as described below.

Approximately 30 mg of maize leaf tissue was harvested from germinating seedlings using forceps and scissors to cut the tip of the second youngest leaf (V2), 7 days after planting. DNA was purified using the BioSprint 96 DNA Plant Kit following the manufacturer’s instructions (Qiagen). A Quant-iT™ PicoGreen™ dsDNA Asasy (Invitrogen) was run to quantify DNA. Samples were normalized to 5 ng/μL for qPCR template.

Zygosity of the *aad-1* transgene was determined by qPCR in a duplex reaction with invertase as the internal control. The total reaction volume was 5.0 μL and contained the following: 2.5 μL LightCycler® 480 Probes Master mix (Roche), 0.3 μL water, 0.2 μL *aad-1* forward and reverse primers [Table 1] (10 μM, 0.2 μL *aad-1* 6FAM-labeled Iowa Black®-quenched probe (5 μM), 0.2 μL IV forward and reverse primers (10 μM), 0.2 μL IV HEX™-labeled Iowa Black®-quenched probe (5 μM) and 1.0 μL DNA (5 ng/μL).

**Table 1:** Primers and probes sequences for genotyping qPCR

Component	Sequence
*aad-1* forward	TGTTCGGTTCCCTCTACCAA
*aad-1* reverse	CAACATCCATCACCTTGACTGA
*aad-1* probe (FAM)	CACAGAACCGTCGCTTCAGCAACA
IV forward	TGGCGGACGACGACTTGT
IV reverse	AAAGTTTGGAGGCTGCCGT
IV probe (HEX)	CGAGCAGACCGCCGTGTACTTCTACC

The assay was ran on Roche LightCycler® 480 Instrument II system under the following conditions: initial 95 °C activation for 10 min, forty cycles of 95 °C denaturation for 10 s, 60 °C anneal for 40 s, 72 °C extension for 1 s, and 40 °C final cooling for 10 s. Reactions for each sample were set up in triplicate. A target to reference ratio was calculated using the comparative cycle threshold (Ct) method known as delta delta Ct (ΔΔCt) to determine zygosity of each sample.

### Transcript abundance analysis for aad-1

Total RNA from flash frozen leaf or root samples was isolated using the MagMAX™-96 Total RNA Isolation Kit (Life Technologies) and a Tecan automated liquid handler. This process includes a DNase treatment step. RNA was quantified using a NanoDrop 8000 Spectrophotometer (ThermoScientific) and, if necessary, concentration was adjusted to less than 50 ng/μL. Quality of RNA was determined by spectroscopy as measured by A260/A280 falling within a range of 2.1 ± 0.2. First strand cDNA was synthesized following manufacturer’s instructions using the High-Capacity cDNA Reverse Transcription Kit (Invitrogen) in a 10 μL reaction containing 5 μL of total RNA. Following synthesis, cDNA was diluted 1:3 with nuclease free water and stored at −20 °C until ready for qPCR assay. Quantitative PCR assays were set up using the ep*Motion*® 5075 liquid handler (Eppendorf). Each sample was assayed in a 384-well plate in triplicate for target gene (*aad-1*) and two reference gene assays depending on the tissue type [Tables 2, 3]. Primers and Roche Universal Probe Library (UPL) probes for the RT-qPCR assays can be found in the Additional file section [Additional file 4]. Each well contained 4 μL of assay mix and 1 μL of cDNA. Assay mix consisted of forward and reverse primer at a final concentration of 0.25 μM and UPL probe at a final concentration of 0.1 μM with 1× LightCycler® 480 Probes Master mix. The 6FAM channel was used for detection in both assays. PCR cycling conditions were initial activation at 95 °C for 10 min followed by 43 cycles of denaturation at 95 °C for 10 s, annealing and extension at 60 °C for 20 s and data acquisition for 1 s at 72 °C. Assay plates were run on the Roche LightCycler® 480 Instrument II system and the mean crossing point (C_p_) cycle for the triplicate runs was calculated for each sample prior to further statistical analysis. Relative transcript abundance was calculated as transcript-to-reference (T/R) values derived by the formula ${2}^{-\Big({C}_P Target-\sqrt[2]{C_p Ref1\times {C}_p\mathit{\operatorname{Re}}f2\Big)}}$, where *C*_*P*_*Target* is the cycle crossing point of *aad-1* driven by the target promoter and $\sqrt[2]{C_p Ref1\times {C}_p Ref2}$ is the geometric mean of the cycle crossing points for the two reference genes [30].

**Table 2:** Reference genes used for transcript abundance analysis

Assay	Target	Accession #	Comments
TIP	TIP41-like	BT069734	Maize homologue to Arabidopsis TIP41-like [31]
MAZ95	Actin	U60507	Root preferred Actin [32].
GDH	GAPDH	X15596	Glyceraldehyde-3′-phosphate dehydrogenase [33]
EFA	eEF1-alpha	AF136823	Elongation factor [34]
SUP	Sal1	AY243475	Supernumerary aleruone layer gene [35]

**Table 3:** Reference assays used for transcript accumulation by tissue/stage

Maize Tissue	Dev Stage	Ref1	Ref [2]
Root	V3	SUP	MAZ95
Leaf	V3	SUP	TIP
Leaf	V8	SUP	TIP
Tassel	R1	GDH	TIP
Silk	R1	GDH	EFA
Husk	R1	SUP	TIP

### AAD-1 protein quantification

#### Protein extraction and AAD-1 ELISA

Extractions were carried out using phosphate buffered saline tween (PBST) buffer supplemented with 0.05% BSA (Serological Corporation). Samples were bead beat with one 3.2 mm stainless steel bead at a rate of 500 at 1× (1500) strokes per minute for 5 min using a Geno/Grinder® 2000 (SPEX® SamplePrep) and centrifuged for 5 min at 1449 rcf (xg). Supernatant was collected and used for assay.

Customized AAD-1 ELISA kits (Envirologix Portland, ME) were used for quantification of AAD-1 protein. The kits contained plates pre-coated with a monoclonal anti AAD-1 antibody, a biotinylated monoclonal AAD-1 primary detection antibody, a Streptavidin/Alkaline Phosphatase secondary detection antibody and the Alkaline Phosphatase PNPP (*p*-Nitrophenyl Phosphate, Disodium Salt) substrate. A seven point standard curve, from 200 ng/ml to 3.125 ng/ml, using AAD-1 microbial protein produced at Dow AgroSciences LLC was included for quantiation.

A total of 100 μl per well of samples or standards were added to the AAD-1 ELISA plate along with 50 μL per well of the biotinylated mouse anti-AAD-1. Plates were sealed and placed on a shaker at a speed of 250 rpm (Labline Instruments; Titer Plate Shaker) at room temperature for 1 h. Plates were then washed 4 times with 300 μL per well of wash buffer (WB) containing PBS supplemented with 0.05% Tween® 20 using a plate washer (Tomtec; Quadra Wash 2). Diluted Streptavidin/Alkaline Phosphatase solution was added to plates at 100 μl per well. Plates were again sealed and incubated at room temperature for 30 min with shaking. The plates were washed as before and 100 μL per well of alkaline phosphate substrate (PNPP) was added. Plates were sealed and incubated at room temperature for 30 min with shaking. Absorbance was read at 405 nm with an optional 650 nm reference using a 96 well plate reader (Molecular Devices SpectraMax® 340pc).

#### Data transformation and analysis

The protein quantification and relative transcript abundance data were log2 transformed and analyzed by tissue (e.g. root) and stage (e.g. V8) according to the model: *γ*_*ij*_ = *μ* + *ρ*_*i*_ + *τ*_*j*(*i*)_ + *ε*_*ij*_, where y represents the log2-transformed measurment on a sample; *ρ*_*i*_ is the fixed effect of the *i*th promoter; *τ*_*j*_ is the random effect of the *j*th transgene integration event within the *i*th promoter; and *ε*_*ij*_ is the residual error. The ordinal herbicide tolerance rankings were not transformed, but they were similarly treated as a continuous linear response using the same model as the transcript data in order to facilitate general comparisons against the protein and transcript results. The herbicide response data were modeled separately for each number of days after application (e.g. 7 DAA) and herbicide application rate (e.g. 1120 g ae ha^−1^).

The models were fit by restricted estimate maximum likelihood (REML) in version 3.3.2 of R [36] using the lmer function from the R package lme4 [37]. Marginal and conditional R^2^ values of the models were assessed with the sem.model.fits function of the R package piecewiseSEM (Additional file 5) [38]. The least square means for the promoter effect of the fit model were estimated in R with the lsmeans function, wherein degrees of freedom were calculated with the kenward-roger method, of the R package lsmeans [39], and significant (alpha = 0.05) differences between these means were determined with the cld function of the R package multcompView [40]. The figures were generated using the R package ggplot2 [41].

#### Herbicide tolerance analysis

T1 events for each construct were chosen based on previous germination and expression analyses. Maize seeds were planted at a depth of 1 in. into 4 in. pots filled with Metro Mix® 360 media. Plants were germinated in a glasshouse (27 °C, 50 ± 30% RH, 16 h light: 8 h dark, minimum 500 μE/m^2^s^1^ natural + supplemental light) conditions. Germinated plants were sampled at the V2 leaf stage for genotyping of the *aad-1 gene*. In the T1 generation, it is expected that events would be segregating 1:1 for the *aad-1 gene*.

Four replicates of each event were then separated and a herbicide dose response of quizalafop-P-ethyl (0.88 lb. ai gal^−1^ quizalofop P-ethyl, DuPont Chemical Company, Wilmington, DE) was applied using a track sprayer with a fan tip nozzle (8002E flat fan, Tee Jet Technologies, Wheaton, IL) set to deliver a rate of 187 L ha^−1^. The commercial mixture of quizalafop-P-ethyl was formulated with 1% *v*/v Agri-dex (crop oil concentrate, Helena Chemical Company, Collierville, TN). The herbicide applications were made at the V3 growth stage. The application rates of quizalafop-P-ethyl begin at 280 g ae ha^−1^, which is approximate to a 4X use rate, where 1X is the rate of herbicide applied under field conditions to control monocot weed species. These elevated rates were specifically chosen in order to challenge the metabolism of the AAD-1 protein. It should be noted by the reader that rates of 4X and above, as applied in this study, are not relevant to the commercial assessment of the traits but used as a tool to detect differences in the tolerance provided by the promoter and gene of interest. Visual assessments were made at 7 and 14 DAA (days after application) for chlorosis, overall injury and height measurements to calculate growth inhibition from the untreated controls (14 DAA only).

## Additional files

Additional file 1: Transcript abundance by RT-qPCR data. (CSV 90 kb)

Additional file 2: Protein accumulation by ELISA data. (CSV 29 kb)

Additional file 3: Herbicide tolerance by visual injury scoring data. (CSV 18 kb)

Additional file 4: Herbicide tolerance data, list of primers and UPL probes used in RT-qPCR assays. Table listing all the primers and UPL probes used for transcript abundace data. (DOCX 12 kb)

Additional file 5: Model Summary Table. The sample size (n), Akaike information criterion (AIC) and marginal and conditional R2 values are given for each measurement on each sample type (combination of stage and tissue or combination of observation day and herbicide application rate). The overall *p*-value of the fixed construct effect is also shown. (DOCX 13 kb)

## Abbreviations

AAD-1: aryloxyalkanoate dioxygenase
CmActin: Melon actin
Ct: Cycle threshold
DAA: Days after application
DAP: Days after planting
ELISA: Enzyme linked immunosorbent assay
g ae ha^−1^: grams equivalent per hectare
I: Intron
IM: Irene Maleng genome
OsActin: Rice actin
OsUbi: Rice ubiquitin
PBST: Phosphate buffered saline tween
PNPP: Alkaline phosphate substrate
qPCR: Quantitative PCR
R1: First reproductive growth stage in maize
R2: Second reproductive growth stage in maize
REML: Restricted estimate maximum likelihood
RT-qPCR: Reverse transcriptase quantitative polymerase chain reaction
SCBV: Sugarcane bacilliform badnavirus
SCBV-IM + I: Sugarcane bacilliform badnavirus irene maleng genome plus intron
SCBV-IM: Sugarcane bacilliform badnavirus irene maleng genome
SCBV–UM: Sugarcane bacilliform badnavirus mor genome from university of minessota
UM: Mor genome from university of minnesota
UPL: Universal probe library
V3: Third vegetative growth stage in maize
V8: Eighth vegetative growth stage in maize
ZmUbi: Maize ubiquitin promoter
ΔΔCt: Delta Delta Ct

## References

[1] Duke SO (2015). Perspectives on transgenic, herbicide-resistant crops in the United States almost 20 years after introduction. Pest Manag Sci.

[2] Wright TR, Shan G, Walsh TA, Lira JM, Cui C, Song P, Zhuang M, Arnold NL, Lin G, Yau K, Russell SM, Cicchillo RM, Peterson MA, Simpson DA, Zhou N, Ponsamuel J, Zhang Z (2010). Robust crop resistance to broadleaf and grass herbicides provided by aryloxyalkanoate dioxygenase transgenes. Proc Natl Acad Sci U S A.

[3] Potenza C, Lorenzo A, Champa SG (2004). Invited review: targeting transgene expression in research, agricultural, and environmental applications: promoters used in plant transformation. Vitr Cell Dev Biol Plant.

[4] Kim JH, Zhang D, Kim HY (2014). Detection of sixteen genetically modified maize events in processed foods using four event-specific pentaplex PCR systems. Food Control.

[5] Mol JN, Stuitje AR, Krol A (1989). Genetic manipulation of floral pigmentation genes. Plant Mol Biol.

[6] Park YD, Papp I, Moscone E, Iglesias V, Vaucheret H, Matzke A, Matzke M (1996). Gene silencing mediated by promoter homology occurs at the level of transcription and results in meiotically heritable alterations in methylation and gene activity. Plant J.

[7] Mette M, Van der Winden J, Matzke M, Matzke A (1999). Production of aberrant promoter transcripts contributes to methylation and silencing of unlinked homologous promoters in trans. EMBO.

[8] Christensen AH, Sharrock RA, Quail PH (1992). Maize polyubiquitin genes: structure, thermal perturbation of expression and transcript splicing and promoter activity following transfer to protoplast by electroporation. Plant Mol Biol.

[9] Sivamani E, Qu R (2006). Expression enhancement of a rice polyubiquitin promoter. Plant Mol Biol.

[10] Clendennen S.K, Kellogg JA, Phan CB, Mathews HV, Webb NM. Melon promoters for expression of transgenes in plants. 2003; US Patent No. 6,642,438.

[11] McElroy D, Zhang WG, Cao J, Wu R (1990). Isolation of an efficient Actin promoter for use in rice transformation. Plant Cell.

[12] Braithwaite KS, Geijskes RJ, Smith GR (2004). A variable region of the sugarcane bacilliform virus (SCBV) genome can be used to generate promoters for transgene expression in sugarcane. Plant Cell Rep.

[13] Tzafrir I, Torbert KA, Lockhart BEL, Somers DA, Olszewski NE (1998). The sugarcane bacilliform badnavirus promoter is active in both monocots and dicots. Plant Mol Biol.

[14] Christensen AH, Quail PH (1996). Ubiquitin promoter-based vectors for high-level expression of selectable and/or screenable marker genes in monocotyledonous plants. Transgenic Res.

[15] Cornejo MJ, Luth D, Blankenship KM, Anderson OD, Blechl AE (1993). Activity of a maize ubiquitin promoter in transgenic rice. Plant Mol Biol.

[16] Liu D, Oard SV, Oard JH (2003). High transgene expression levels in sugarcane (Saccharum officinarum L.) driven by the rice ubiquitin promoter RUBQ2. Plant Sci.

[17] GKA P, BAHARIAH B, Ayub NH, AMM Y, Rasid OA, Hashim AT (2008). Transformation of PHB and PHBV genes driven by maize ubiquitin promoter into oil palm for the production of biodegradable plastics. J Oil Palm Res.

[18] Ritchie SW, Hanway JJ, Benson GO, Herman JC, Lupkes SJ. How a corn plant develops. Iowa State university of Science and Technology Coop. Ext. Services Ames, Iowa. 1993; Special report, 48.

[19] Klümper W, Qaim M (2014). A meta-analysis of the impacts of genetically modified crops. PLoS One.

[20] Martino-Catt SJ, Sachs ES (2008). The next generation of biotech crops. Plant Physiol.

[21] Lombardo L, Coppola G, Zelasco S (2016). New technologies for insect-resistant and herbicide-tolerant plants. Trends Biotechnol.

[22] Rose AB. Intron-mediated regulation of gene expression. Nucl Pre-mRNA Proc Plants. 2008:277–90. 10.1007/978-3-540-76776-3_15 18630758

[23] Gallegos JE, Rose AB (2015). (2015) the enduring mystery of intron-mediated enhancement. Plant Sci.

[24] Vain P, Finer KR, Engler DE, Pratt RC, Finer JJ (1996). Intron-mediated enhancement of gene expression in maize (Zea Mays L.) and bluegrass (Poa Pratensis L.). Plant Cell Rep.

[25] Salgueiro S, Pignocchi C, Parry MA (2000). Intron-mediated gusA expression in tritordeum and wheat resulting from particle bombardment. Plant Mol Biol.

[26] Wang Y, Lang Z, Zhang J, He K, Song F, Huang D (2008). Ubi1 intron-mediated enhancement of the expression of Bt cry1Ah gene in transgenic maize (Zea Mays L.). Chin Sci Bull.

[27] Davies JP, Reddy V, Liu XL, Reddy AS, Ainley WM, Thompson M, Sastry-Dent L, Cao Z, Connell J, Gonzalez DO, Wagner DR (2014). Identification and use of the sugarcane bacilliform virus enhancer in transgenic maize. BMC Plant Biol.

[28] Ishida Y, Saito H, Ohta S, Hiei Y, Komari T, Kumashiro T (1996). High efficiency transformation of maize (*Zea mays L*.) mediated by *Agrobacterium tumefaciens*. Nat Biotech.

[29] Frame BR, McMurray JM, Fonger TM, Main ML, Taylor KW, Torney FJ, Paz MM, Wang K (2006). Improved Agrobacterium mediated transformation of three maize inbred lines using MS salts. Plant Cell Rep.

[30] Vandesompele J, De Preter K, Pattyn F, Poppe B, Van Roy N, De Paepe A, Speleman F (2002). Accurate normalization of real-time quantitative RT-PCR data by geometric averaging of multiple internal control genes. Genome Biol.

[31] Czechowski T, Stitt M, Altmann T, Udvardi MK, Scheible WR (2005). Genome-wide identification and testing of superior reference genes for transcript normalization in Arabidopsis. Plant Physiol.

[32] Moniz de Sá M, Drouin G (1996). 1996 Phylogeny and substitution rates of angiosperm Actin genes. Mol Biol Evol.

[33] Martinez P, Martin W, Cerff R (1989). Structure, evolution and anaerobic regulation of a nuclear gene encoding cytosolic glyceraldehyde-3-phosphate dehydrogenase from maize. J Mol Biol.

[34] Carneiro NP, Hughes PA, Larkins BA (1999). The eEFIA gene family is differentially expressed in maize endosperm. Plant Mol Biol.

[35] Shen B, Li C, Min Z, Meeley RB, Tarczynski MC, Olsen OA (2003). sal1 determines the number of aleurone cell layers in maize endosperm and encodes a class E vacuolar sorting protein. Proc Natl Acad Sci U S A.

[36] R Development Core Team. R (2015). A language and environment for statistical computing (version 3.2.1) [software].

[37] Bates D, Maechler M, Bolker B, Walker S. Fitting Linear Mixed-Effects Models Using lme4. R package version 1.1-10. J Stat Software. 2015;67(1):1–48. doi:10.18637/jss.v067.i01

[38] Lefcheck JS (2015). piecewiseSEM: piecewise structural equation modeling in R for ecology, evolution, and systematics. Methods Ecol Evol.

[39] Lenth RV. Least-Squares Means: The R Package lsmeans. J Stat Software. 2016;69(1):1–33. doi:10.18637/jss.v069.i01.

[40] Graves S, Piepho HP, Selzer L with help from Sundar Dorai-Raj. multcompView: Visualizations of Paired Comparisons. 2015; R package version 0.1-7. http://CRAN.R-project.org/package=multcompView.

[41] Wickham H. ggplot2: elegant graphics for data analysis. R package version 2.0.0. Springer-Verlag: New York; 2009.

