# Supplementary material for: Expression characterization of the herbicide tolerance gene Aryloxyalkanoate Dioxygenase (aad-1) controlled by seven combinations of regulatory elements
Source: BMC Plant Biol. 2018 Jan 15;18:14. doi: 10.1186/s12870-018-1227-3 (PMC5769356; doi:10.1186/s12870-018-1227-3)
Supplement: Supplementary file 4 — Herbicide tolerance data, list of primers and UPL probes used in RT-qPCR assays. Table listing all the primers and UPL probes used for transcript abundace data. (DOCX 12 kb) [file 12870_2018_1227_MOESM4_ESM.docx]

**Additional File 4**

List of primers and UPL probes used in RT-qPCR assays

| **TARGET** | **NAME** | **SEQUENCE** | **TYPE** |
| --- | --- | --- | --- |
| *aad-1* | 111U53S | aaccatgcaagccaccat | primer |
|  | 111U53A | ggtagagggaaccgaacaca | primer |
|  | UPL53 | ctctgcca | probe |
| TIP | MZTIPU67F | agccaagccagtggtacttc | primer |
|  | MZTIPU67R | tcgcagacaaagtagcaaatgt | primer |
|  | UPL67 | tgctggag | probe |
| MAZ95 | MAZ95U9F | gctgtcctttccctgtatgc | primer |
|  | MAZ95U9R | gcgcgtatccctcgtagat | primer |
|  | UPL9 | tggtgatg | probe |
| Maize GAPDH | MZGDHU142F | cgctgagtacgtcgtggag | primer |
|  | MZGDHU142R | gcttggggcagagataacaa | primer |
|  | UPL142 | ttcttggc | probe |
| Maize eEF1-alpha | MZEFAU22F | tgaagatgatacccaccaagc | primer |
|  | MZEFAU22R | ctacccaggggaggatacg | primer |
|  | UPL22 | ctccacca | probe |
| SUP | REF3U10F | caggccgagttcatggag | primer |
|  | REF3U10R | tgcatgaggctgttgacct | primer |
|  | UPL10 | ccacctcc | probe |
