# Supplementary material for: Expression characterization of the herbicide tolerance gene Aryloxyalkanoate Dioxygenase (aad-1) controlled by seven combinations of regulatory elements
Source: BMC Plant Biol. 2018 Jan 15;18:14. doi: 10.1186/s12870-018-1227-3 (PMC5769356; doi:10.1186/s12870-018-1227-3)
Supplement: Supplementary file 5 — Model Summary Table. The sample size (n), Akaike information criterion (AIC) and marginal and conditional R2 values are given for each measurement on each sample type (combination of stage and tissue or combination of observation day and herbicide application rate). The overall p-value of the fixed construct effect is also shown. (DOCX 13 kb) [file 12870_2018_1227_MOESM5_ESM.docx]

**Additional File 5**

| Model Summary. The sample size (n), Akaike information criterion (AIC) and marginal and conditional R2 values are given for each measurement on each sample type (combination of stage and tissue or combination of observation day and herbicide application rate). The overall p-value of the fixed construct effect is also shown. | | | | | | |
| --- | --- | --- | --- | --- | --- | --- |
| Measurement | Sample Type | n | Marginal R2 | Conditional R2 | AIC | Construct P-value |
| Transformed Expression Ratios | V3 Root | 254 | 0.55 | 0.77 | 881 | 2.89E-06 |
|  | V3 Leaf | 248 | 0.74 | 0.98 | 484 | 5.76E-08 |
|  | V8 Leaf | 82 | 0.79 | 0.95 | 157 | 1.04E-05 |
|  | R1 Tassel | 74 | 0.60 | 0.96 | 121 | 9.62E-03 |
|  | R1 Silk | 71 | 0.54 | 0.96 | 141 | 4.04E-03 |
|  | R1 Husk | 71 | 0.54 | 0.96 | 141 | 1.22E-02 |
|  |  |  |  |  |  |  |
| Transformed Protein Abundance | V3 Root | 67 | 0.3 | 0.44 | -4.08 | 5.64E-02 |
|  | V3 Leaf | 69 | 0.78 | 0.9 | -77.2 | 1.41E-04 |
|  | V8 Leaf | 71 | 0.34 | 0.34 | 14.3 | 3.58E-03 |
|  | R1 Tassel | 56 | 0.61 | 0.79 | 16.2 | 1.04E-02 |
|  | R1 Silk | 63 | 0.56 | 0.94 | -53.6 | 3.19E-02 |
|  | R1 Husk | 61 | 0.6 | 0.91 | -25.7 | 1.03E-02 |
|  |  |  |  |  |  |  |
| Injury Rating | 7DAA of 280 | 58 | 0.57 | 0.88 | 252 | 1.15E-02 |
|  | 7DAA of 560 | 66 | 0.55 | 0.73 | 357 | 2.54E-03 |
|  | 7DAA of 1120 | 68 | 0.66 | 0.85 | 380 | 3.08E-04 |
|  | 14DAA of 280 | 58 | 0.51 | 0.73 | 260 | 9.64E-03 |
|  | 14DAA of 560 | 66 | 0.48 | 0.81 | 334 | 7.25E-03 |
|  | 14DAA of 1120 | 68 | 0.54 | 0.68 | 370 | 1.50E-03 |
